# Unusual flow patterns in a left ventricular aneurysm in hypertrophic cardiomyopathy: a case report

**DOI:** 10.1093/ehjcr/ytag108

**Published:** 2026-02-09

**Authors:** Salman Salahuddin, Bijoy Karunakaran

**Affiliations:** Department of Cardiology, Aster MIMS Hospital, Mini Bypass Road, Govindapuram, Kerala, Calicut 673016, India; Department of Cardiology, Aster MIMS Hospital, Mini Bypass Road, Govindapuram, Kerala, Calicut 673016, India

**Keywords:** Hypertrophic cardiomyopathy, Apical aneurysm, Paradoxical Doppler, Contrast echocardiography, Case report

## Abstract

**Background:**

Apical aneurysms are a recognized complication of hypertrophic cardiomyopathy (HCM), reported in up to 30% of cases with apical and mid-ventricular involvement. They carry significant arrhythmic and thrombo-embolic risk, but the spectrum of associated intracavitary flow abnormalities is not fully described.

**Case summary:**

An 86-year-old woman presented with ventricular tachycardia, successfully cardioverted to sinus rhythm. Echocardiography demonstrated preserved ejection fraction with severe mid-ventricular and apical hypertrophy, and a large apical aneurysm (5 cm) confirmed on contrast echocardiography without thrombus. Global longitudinal strain was reduced (−5.6%). Cardiac magnetic resonance revealed mid-cavity hypertrophy and aneurysm formation, with patchy late gadolinium enhancement at the right ventricular septal insertion site, predominantly in the basal and mid-anteroseptal walls. Doppler interrogation identified a novel bifid systolic jet directed into the aneurysm during isovolumic contraction, as well as a paradoxical early diastolic jet from apex to base. Colour Doppler showed swirling intra-aneurysmal flow with a Yin–Yang appearance. The patient was stabilized on antiarrhythmics and advised implantable cardioverter-defibrillator therapy.

**Discussion:**

While paradoxical diastolic flow has been previously described in HCM with mid-ventricular obstruction, paradoxical bifid systolic jets have rarely been reported. These findings likely reflect transient pressure gradients between the contracting ventricle and the compliant aneurysm. The Yin–Yang Doppler pattern, more commonly associated with pseudoaneurysms, here reflected abnormal haemodynamics within a true aneurysm. This case highlights the importance of multimodality imaging not only for structural characterization and thrombus exclusion but also for identifying atypical intracavitary flow dynamics that may expand understanding of HCM pathophysiology.

Learning points

1. Apical aneurysms in HCM are associated with an increased risk of ventricular arrhythmias and thromboembolism.
2. Unusual intracavitary Doppler flow patterns should be recognized in HCM to avoid misinterpretation.
3. Apical aneurysm represents an important marker of arrhythmic risk beyond the HCM Risk-SCD score.

## Introduction

Hypertrophic cardiomyopathy (HCM) is a genetically determined myocardial disorder characterized by asymmetric hypertrophy of the left ventricle in the absence of another loading condition. Among its diverse phenotypes, apical and mid-ventricular HCM represents distinct morphological variants, where hypertrophy predominantly involves the apex and mid-cavity of the left ventricle. Chronic apical pressure overload and microvascular ischaemia can lead to apical thinning and aneurysm formation, a pathophysiological evolution seen in up to 30% of apical and mid-ventricular HCM cases in some series. The formation of these aneurysms marks a transition from a compensated hypertrophic phase to one of adverse remodelling and fibrotic scarring.

Apical aneurysms in HCM are clinically significant because they act as substrates for ventricular arrhythmias, thrombus formation, and embolic events. The thin, akinetic or dyskinetic aneurysmal segment often harbours fibrosis and slow conduction pathways predisposing to malignant ventricular tachyarrhythmias and sudden cardiac death. Furthermore, the stagnant flow within the aneurysmal cavity favours mural thrombus formation, conferring an increased risk of stroke and systemic embolism, even in the absence of atrial fibrillation. Recognition of these aneurysms is thus critical for risk stratification, anticoagulation decisions, and planning of device therapy.

In this report, we describe an unusual pattern of intra-aneurysmal flow dynamics identified in a patient with apical and mid-cavitary hypertrophic cardiomyopathy complicated by an apical aneurysm.

## Summary figure

**Figure:**
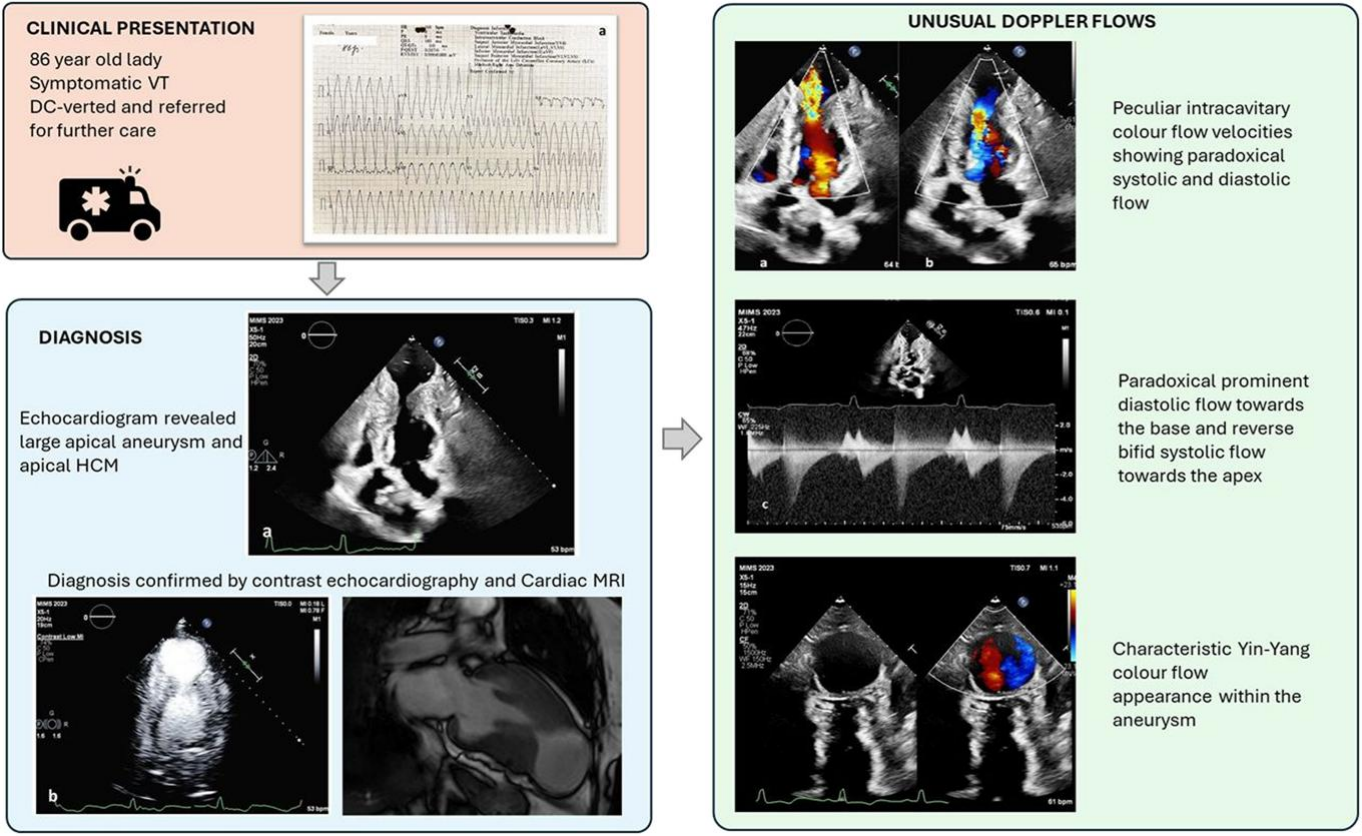


## Case presentation

**Figure 1:**
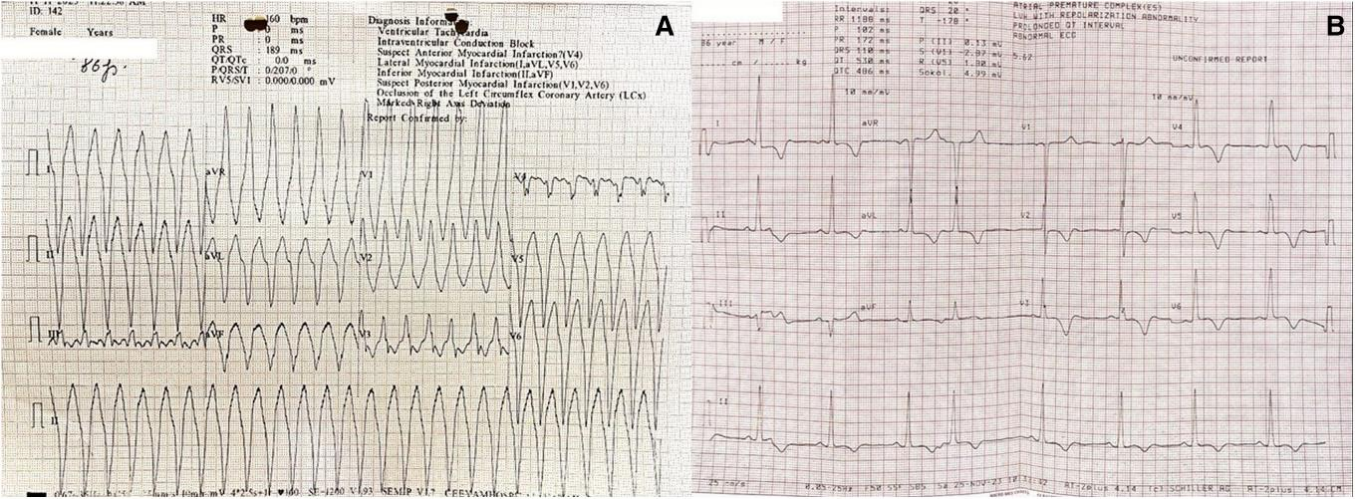
*(A*) Wide QRS tachycardia of 160 b.p.m. suggestive of monomorphic ventricular tachycardia. *(B*) After DC version, baseline ECG shows normal sinus rhythm with left ventricular hypertrophy with strain.

An 86-year-old woman presented to a peripheral hospital with palpitations and giddiness. The 12-lead electrocardiogram demonstrated broad QRS tachycardia at 160 b.p.m. consistent with ventricular tachycardia (*Figure 1A*). She was successfully cardioverted to sinus rhythm with 100 J synchronized DC shock and referred to our centre. On admission, her ECG showed sinus rhythm with left ventricular hypertrophy and strain pattern (*Figure 1B*).

**Figure 2:**
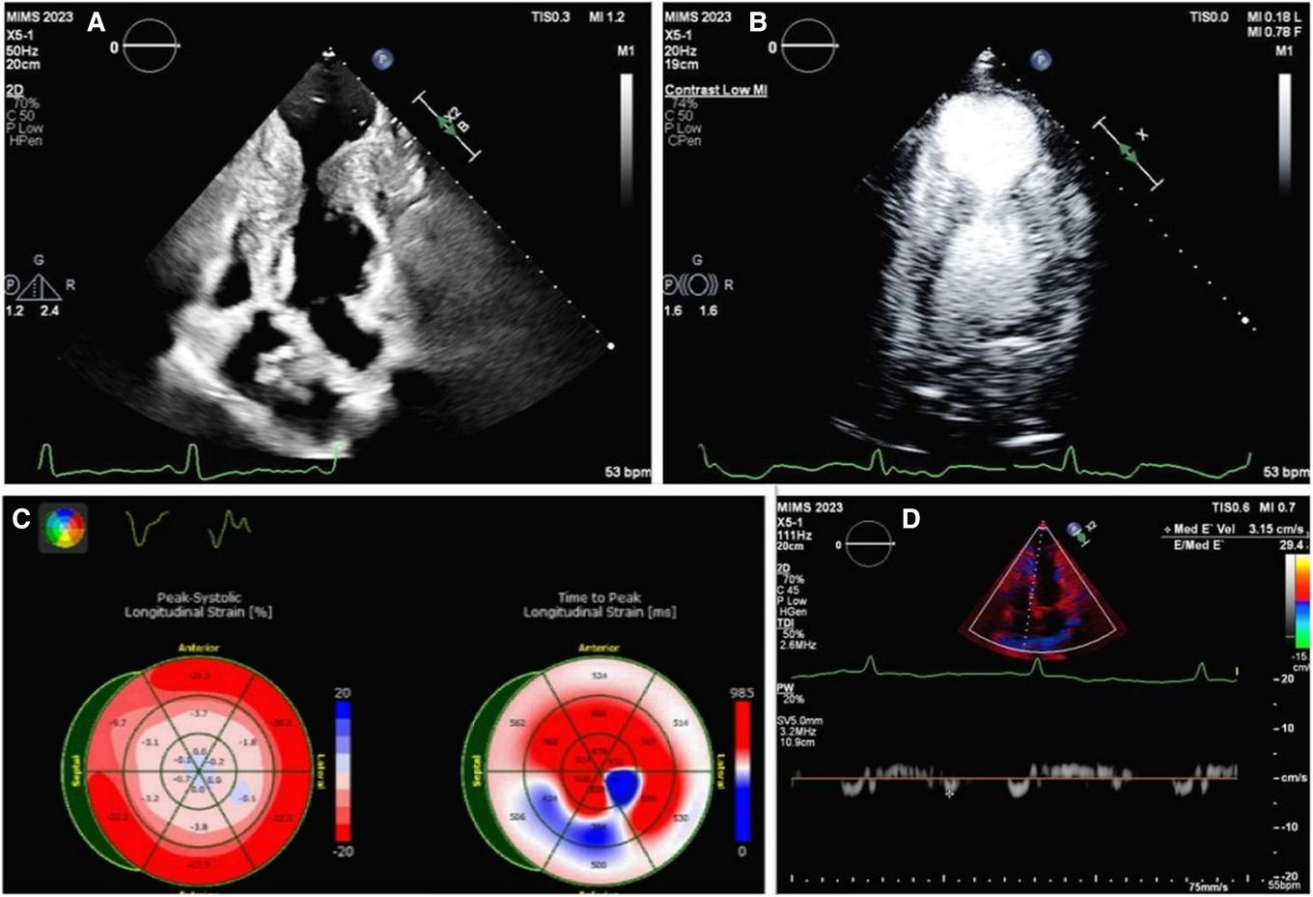
*(A*) 2D echocardiogram in the apical four-chamber view showing the apical aneurysm and the apical and mid-septal left ventricular hypertrophy; *(B)* contrast echocardiography delineating the large apical aneurysm and confirming no thrombus; *(C)* global longitudinal strain analysis showing decreased apical strain values; *(D)* low mitral annular tissue Doppler velocity of 3.1 cm/s.

**Figure 3:**
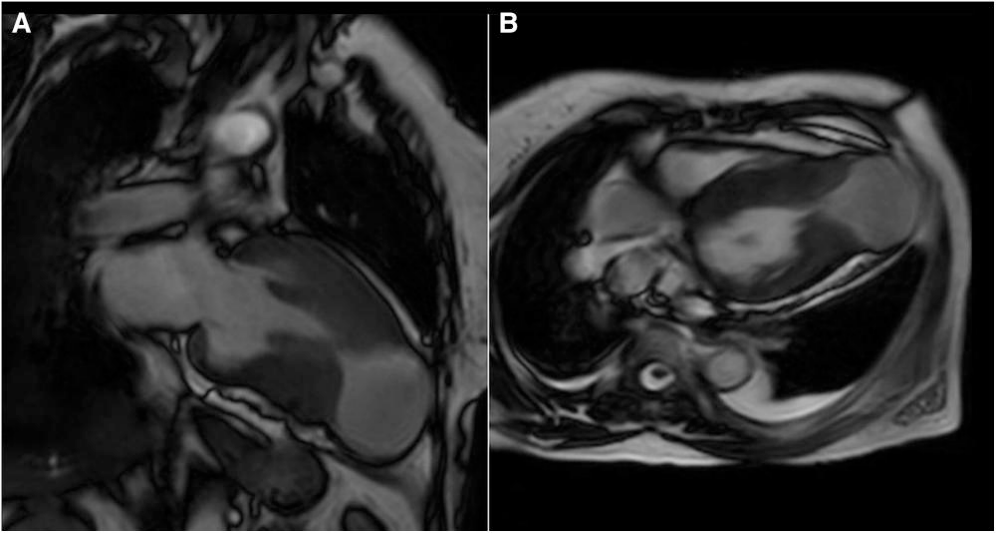
Cardiac MRI demonstrating the apical aneurysm and apical and mid-septal hypertrophy in *(A)* two-chamber and *(B)* four-chamber long-axis views.

Transthoracic echocardiography revealed preserved left ventricular systolic function with marked hypertrophy in the apical and mid-ventricular segments. A large apical outpouching, severely hypokinetic and measuring 5 cm, was noted (*Figure 2A*) (see Supplementary material online, *Video S1*). Contrast echocardiography (SonoVue®, Bracco) confirmed a large circular apical aneurysm without thrombus (*Figure 2B*) (see Supplementary material online, *Video S2*). The systolic function appeared to be normal; however, the global longitudinal strain was severely reduced (−5.6%) with very low apical longitudinal strain (*Figure 2C*). Cardiac MRI (CMR) demonstrated prominent mid-cavity hypertrophy, apical outpouching (5 cm in diameter), and patchy late gadolinium enhancement (LGE) at the right ventricular septal insertion site, predominantly in the basal and mid-anteroseptal walls (*Figure 3*). A diagnosis of hypertrophic cardiomyopathy with mid-ventricular and apical involvement with an apical aneurysm was made. Coronary angiography revealed no significant obstructive coronary artery disease.

**Figure 4:**
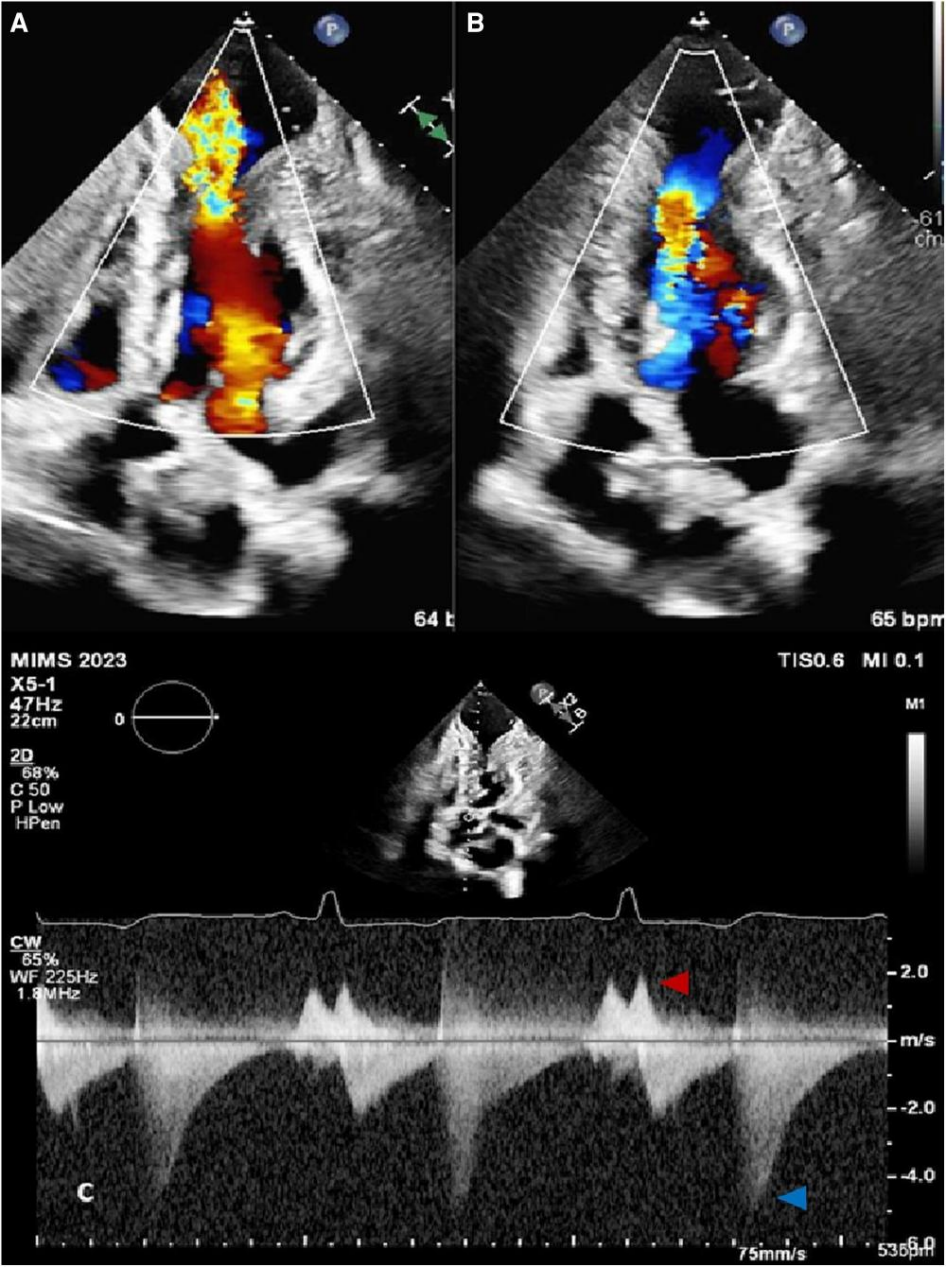
*(A*) Colour Doppler showing paradoxical systolic flow into the apical aneurysm, and *(B)* paradoxical diastolic flow from apex to base. *(C*) Continuous wave Doppler revealing paradoxical bifid systolic flow (red arrow), and prominent reverse diastolic flow of 5 m/s towards the base (blue arrow).

**Figure 5:**
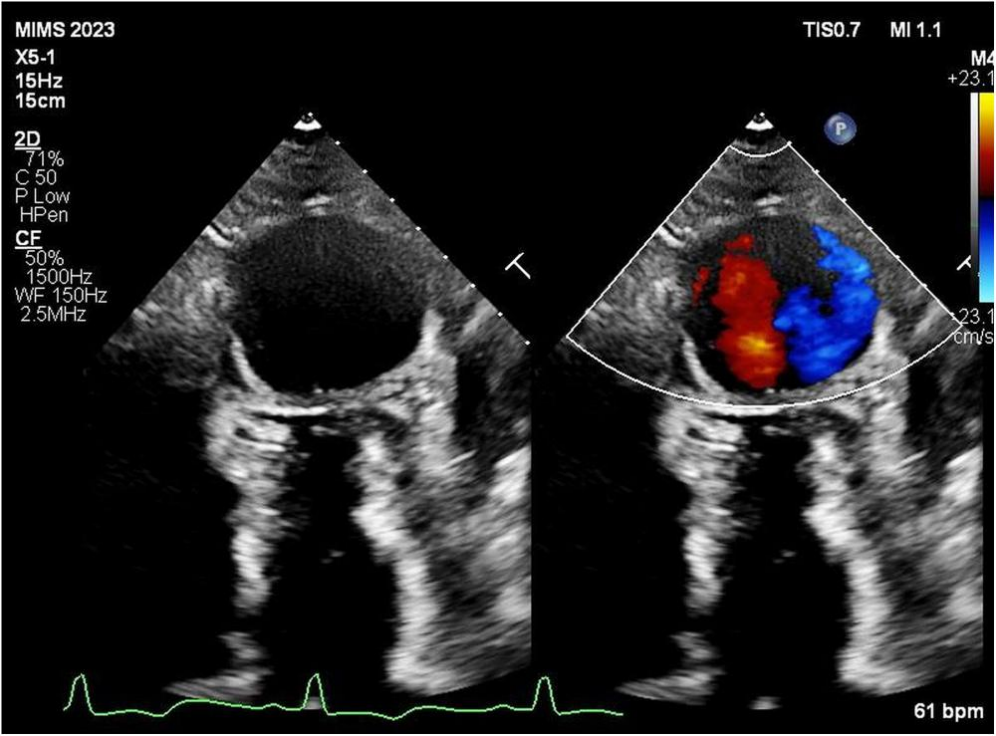
Colour flow Doppler imaging showing swirling flow within the aneurysm—the typical ‘Yin–Yang sign’.

On detailed Doppler assessment, several unusual intraventricular flow patterns were observed. A paradoxical systolic jet, bifid in character, was seen directed apically into the aneurysm, during isovolumic contraction (*Figure 4*). No significant LV outflow tract obstruction was present. Mitral inflow showed a pseudo-normal pattern (E/A 1.4), with reduced annular *e*′ velocity (3 cm/s), consistent with grade II diastolic dysfunction (*Figure 2D*). Remarkably, a paradoxical diastolic flow was recorded from the aneurysm towards the LV base during early diastole, with a velocity gradient of 5 m/s (*Figure 4*). Colour Doppler further revealed swirling flow within the aneurysm, producing a Yin–Yang pattern typically described in pseudoaneurysms (*Figure 5*).

The patient was stabilized on oral antiarrhythmics and discharged with a recommendation for implantable cardioverter-defibrillator therapy.

## Discussion

Apical aneurysms occur in 2%–5% of hypertrophic cardiomyopathy (HCM) overall, but in 20%–30% of apical and mid-ventricular HCM cohorts.^1,2^ Apical HCM can involve isolated apical hypertrophy or a mixed phenotype with both apical and septal involvement. Aneurysm formation is usually associated with mid-ventricular obstruction and cavity obliteration (MVOCO), more often in mixed variants.^3,4^ Aneurysms are classified by maximum diameter at end-systole in the four-chamber view: Small (<2 cm), medium (2–4 cm), and large (>4 cm).

The mechanisms underlying aneurysm formation in HCM are incompletely understood. The process is thought to progress from apical cavity obliteration to widening of the apical slit, development of an outpouching, and eventual aneurysm formation.^5^ In a longitudinal CMR study, Habib *et al*. identified baseline LV mass, wall thickness, and mid-ventricular gradients as predictors of future aneurysm development.^6^ Although LGE is often expected within apical aneurysms in hypertrophic cardiomyopathy, no significant enhancement was seen in the aneurysm wall in our case, a finding that has also been reported in prior CMR reports of apical aneurysms.^7,8^ A possible explanation may be that if the aneurysm wall is very thin, the signal from gadolinium uptake may be lost due to partial volume averaging with blood pool or adjacent fat.

Sherrid *et al*., in a series of 108 patients with apical aneurysms with HCM, reported several characteristic intracavitary flow patterns. The majority (95%) demonstrated mid-LV obstruction with near-complete systolic emptying.^9^ In our patient with a large apical aneurysm, we documented a clear diastolic jet from apex to base during early diastole. Such diastolic paradoxical flow has been described in HCM with MVOCO and is attributed to elevated apical pressures relative to basal LV pressures during isovolumic relaxation and early diastole. Regional differences in relaxation may accentuate this gradient, with slower apical relaxation compared to mid-cavity segments.^10^ Clinically, paradoxical diastolic flow has been linked to arrhythmia risk, thromboembolism, and adverse outcomes.^11^

In contrast, paradoxical systolic flows have rarely been described in HCM except in case reports.^12^ In our patient, a bifid systolic jet was observed during isovolumic contraction, directed from the LV cavity into the apical aneurysm. This likely reflects transient pressure gradients between the contracting LV and the compliant aneurysm sac. The bifid character may represent interruption by contraction at the aneurysm neck, followed by persistent flow as the pressure gradient continued. To our knowledge, reports of such intraventricular paradoxical biphasic systolic flows have not been described. The additional Yin–Yang sign observed on colour Doppler, a marker of bidirectional swirling flow, is more commonly described in pseudoaneurysms, but here reflected the abnormal haemodynamics within a true aneurysm cavity.

The current HCM Risk-SCD calculator by the European Society of Cardiology remains the standard tool for estimating 5-year sudden cardiac death risk in HCM; however, it does not account for certain high-risk morphological features such as left ventricular apical aneurysm, which is now recognized as a strong and independent predictor of malignant ventricular arrhythmias and adverse outcomes. This limitation underscores the need for future refinements of the risk model to incorporate such structural markers.

This case report has a few limitations. Genetic testing was not performed due to financial constraints. Advanced echocardiographic strain analysis beyond global longitudinal strain, including radial and circumferential strain, was not available. In addition, T2-weighted/STIR sequences were not obtained on cardiac magnetic resonance imaging, limiting assessment of myocardial oedema. Despite these limitations, multimodality imaging adequately characterized the aneurysm and demonstrated the unique intracavitary flow dynamics described.

## Conclusion

Apical aneurysms are a recognized complication of HCM with mid-ventricular obstruction, carrying significant arrhythmic and thrombo-embolic risk. Careful imaging evaluation, including contrast echocardiography and CMR maybe required for diagnosis and exclusion of thrombus. Beyond structural abnormalities, they may serve as a nidus for unusual intra-ventricular flow dynamics. In this case, we describe both paradoxical diastolic base-directed flow and a novel bifid systolic jet into the aneurysm cavity, along with a Yin–Yang Doppler pattern. Awareness of such findings can expand understanding of HCM pathophysiology and aid in risk stratification.

## Lead author biography


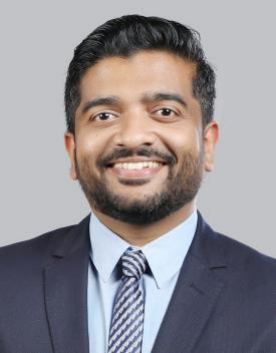


Dr Salman Salahuddin works as an interventional cardiologist at Aster MIMS Hospital, Calicut, India. He leads the Department of Adult Interventional Cardiology. An interventional cardiologist with expertise in complex coronary interventions, structural heart disease, and advanced echocardiography, he is actively involved in teaching, research, and improving patient outcomes.

## Supplementary Material

ytag108_Supplementary_Data

## Data Availability

All relevant data underlying this article are included within the manuscript and its supplementary files. No additional datasets were generated or analysed in the context of this case report.

## Notes

**Consent:** The authors confirm that written informed consent was obtained from the patient for publication of this case report, including any accompanying images and clinical data. The authors affirm that this work complies with the principles outlined by the Committee on Publication Ethics (COPE) and the European Society of Cardiology (ESC) ethical publication guidelines.
